# A proposed lectin‐mediated mechanism to explain the in Vivo antihyperglycemic activity of γ‐conglutin from *Lupinus albus* seeds

**DOI:** 10.1002/fsn3.2520

**Published:** 2021-09-18

**Authors:** Madalena Grácio, João Rocha, Rui Pinto, Ricardo Boavida Ferreira, João Solas, Maria Eduardo‐Figueira, Bruno Sepodes, Ana Cristina Ribeiro

**Affiliations:** ^1^ Faculdade de Farmácia da Universidade de Lisboa Lisboa Portugal; ^2^ Linking Landscape, Environment, Agriculture and Food (LEAF) University of Lisbon Higher Institute of Agronomy Lisbon Portugal; ^3^ Research Institute for Medicines and Pharmaceutical Sciences (iMed.UL) and Faculdade de Farmácia da Universidade de Lisboa Lisboa Portugal; ^4^ JCS Dr Joaquim Chaves Lab Análises Clínicas Algés Portugal; ^5^ HTRC‐Health and Technology Research Center ESTeSL Instituto Superior Técnico Universidade de Lisboa Lisboa Portugal

**Keywords:** diabetes mellitus type 2, hypoglycemic activity, insulin receptors, lectins, γ‐Conglutin

## Abstract

Experiments conducted in vitro and in vivo, as well as clinical trials for hypoglycemic therapeutics, support the hypoglycemic properties of the lectin γ‐conglutin, a *Lupinus* seed storage protein, by a mechanism not yet been clarified.

Structural studies established that binding of γ‐conglutin, in native and denatured form, to insulin occurs by a strong binding that resists rupture when 0.4 M NaCl and 0.4 M galactose are present, suggesting that strong electrostatic interactions are involved. Studies on binding of γ‐conglutin in native and denatured form to HepG2 membrane glycosylated receptors were conducted, which reveal that only the native form of γ‐conglutin with lectin activity is capable of binding to these receptors.

Glycosylated insulin receptors were detected on purified HepG2 cell membranes and characterized by 1D and 2D analyses.

Preclinical assays with male mice (CD‐1) indicated that native and denatured γ‐conglutins display antihyperglycemic effect, decreasing glucose in blood comparable after 120 min to that exhibited by the animal group treated with metformin, used to treat T2D and used as a positive control. Measurement of organ injury/functional biomarkers (hepatic, pancreatic, renal, and lipid profile) was comparable to that of metformin treatment or even better in terms of safety endpoints (pancreatic and hepatic biomarkers).

## Graphical abstract

A lectin mechanism to understanding the antihyperglycemic activity of gamma conglutin. The involved molecules in hypoglycemic activity are majority glycoproteins, excepted insulin.

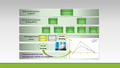

## 1 INTRODUCTION

Goldstein et al. (1980) defined lectin as a "protein or glycoprotein of non‐immune origin (thus excluding immunoglobulins) which bind in a stable manner (thus excluding enzymes and carbohydrate sensor/transport proteins) to carbohydrates.” Plant lectins exert a vast panel of bioactivities (Ribeiro et al., 2018. *Lupinus albus* is a legume whose seeds contain reserve proteins exhibiting an array of bioactivities as wide as insecticide, fungicide (Monteiro et al., 2015), anticancer (Oliveira et al., 2019), and hypoglycemic (Fornasini et al., 2012; González‐Santiago et al., 2017; Magni et al., 2004; Terruzzi et al., 2011). Some of these proteins are lectins, with γ‐conglutin and BLAD (Ribeiro et al., 2014) as examples. Regarding human health benefits, the antitumor activity of many lectins is one of the most studied effects (Oliveira et al., 2019); however, γ‐conglutin has been reported to exert hypoglycemic action (Capraro et al., 2013; Lovati et al., 2011).

γ‐Conglutin is a glycoprotein, with galactose‐specific lectin activity, characteristic of *Lupinus* seeds, with a molecular weight between 37 and 42 kDa, composed of two polypeptide chains each one with some isoforms, and with a basic pI around 8.0 for larger polypeptide and a pI around 6.0 for the smaller polypeptide (Ribeiro et al., 2014).

γ‐Conglutin has been studied regarding its classification as a seed reserve protein (Duranti & Scarafoni, 1995), its structure, and its biological activity in vivo (Sandoval‐Muñíz et al., 2018; Scarafoni et al., 2007), where it is described as having hypoglycemic activity but with no consensus about of its mechanism of action. In in vitro models, it exhibits hypoglycemic activity via glucose reuptake increase by HepG2 cells, with internalization and multiple phosphorylation (Capraro et al., 2013; Lovati et al., 2011), insulin binding (Magni et al., 2004), increased glucose transport GLUT4 translocation in C2C12 myoblastic cells (Terruzzi et al., 2011), and also lowered glucose levels in in vivo hyperglycemic rat models (Magni et al., 2004) and animal models with impaired glucose metabolism (insulin resistance and type 2 diabetes model) (González‐Santiago et al., 2017).

There are many gaps in the knowledge of the hypoglycemic mechanism exhibited by γ‐conglutin in type 2 diabetes mellitus. Some studies reported that γ‐conglutin binds to insulin in an electrostatic‐ dependent manner (Magni et al., 2004), whereas others suggested an insulin‐mimetic action in mouse myoblasts (Terruzzi et al., 2011). Also, the relationship among insulin, insulin receptors, and glucose transporters GLUT2 was studied (Sandoval‐Muñíz et al., 2018) and led the authors to conclude that γ‐conglutin upregulates Slc2a2 gene expression in liver and normalizes GLUT2 protein content in the pancreas of streptozotocin‐induced diabetic rats. In in vitro studies, in which γ‐conglutin hydrolysates were tested in mature 3T3‐L1 adipocytes, they significantly increased GLUT4 translocation to the plasma membrane compared with untreated control cells, meaning that increased glucose uptake was stimulated by *L*. *mutabilis* seed protein hydrolysates, thus indicating that glucose internalization into the cells could be mediated by insulin‐dependent GLUT4 (Muñoz et al., 2018).

Diabetes is a chronic disease caused by metabolic disturbances that leads to an excess of glucose in blood and represents a serious worldwide epidemiologic problem. The aim of this work was to contribute to the improvement of knowledge on the γ‐conglutin mechanism of action based on its lectin properties, which has not yet been described. Our results concerning the antihyperglycemic activity of γ‐conglutin in in vitro (HepG2 cells) and in vivo (mice) models revealed new and promising data. The antihyperglycemic effect, as a result of structural modifications of γ‐conglutin, and its lectin activity on insulin and insulin receptors binding to HepG2 cell membranes as molecular targets were explored. Also, the lectin activity effect on biomarkers of different organ functions was evaluated to complete the antihyperglycemic study. The results obtained suggest that γ‐conglutin in native or denatured form could be a new pharmacologic tool in the management of diabetes and might have a role in the development of new therapies.

## 2 MATERIALS AND METHODS

### 2.1 Plant material and animal cells

#### 2.1.1 Plants

Dry mature seeds from *Lupinus albus* cv amiga were kindly supplied by Dr. J.N. Martins (University of Lisbon, Lisbon, Portugal).

#### 2.1.2 HepG2 cells

HepG2 cell lines of hepatocellular carcinoma (ATCC®HB‐8065™) were used in in vitro assays.

#### 2.1.3 Erythrocyte cells

Rabbit erythrocytes were commercially available (Probiológica).

### 2.2 Seed protein extraction

Seeds from *Lupinus albus* cv amiga were assayed.

#### 2.2.1 Isolation of albumins and total globulins based on solubility criteria

The seeds from *Lupinus albus* cv amiga were powdered, and the meal was defatted with n‐hexane (34 ml/g of flour) for 4 hr with agitation and air‐dried after decantation of the hexane. The albumin and globulin fractions were extracted according Ribeiro et al. (2014).

The albumins were extracted by stirring the powder for 4 hr at 4℃ in water (pH adjusted to 8.0) containing 10 mM CaCl_2_ and 10 mM MgCl_2_ (34 ml/g of dry mass). The insoluble proteins were removed by centrifugation at 30,000 g at 4℃ for 1 hr, and the albumin fraction was retained in the supernatant. For globulin extraction, the pellet was solubilized in 100 mM Tris‐HCl buffer, pH 7.5, containing 10% (w/v) NaCl (34 ml/g of dry mass), and stirred for 4 hr at 4℃. The globulin‐containing solution was centrifuged for 1 hr at 30,000 g, and the globulins remaining in the supernatant were subsequently precipitated by the addition of ammonium sulfate 80%. The precipitated globulins were centrifuged at 30,000 g for 20 min, suspended in 50 mM Tris‐HCl buffer, pH 7.5, and desalted on PD‐10 columns (GE Healthcare Life Sciences), previously equilibrated in the same buffer. All operations were performed at 4℃.

#### 2.2.2 γ‐conglutin purification

A DEAE‐Sepharose matrix was performed in batch to achieve the purification of γ‐conglutin from the globulin fraction. The globulin fraction was mixed with DEAE matrix with a 50 mM Tris‐HCl buffer, pH 7.0 (3.25 g of matrix/5 ml of volume), for 1 hr, at room temperature (25s). The matrix and the globulin fractions were mixed by stirring every 15 min, during 1 hr, to maximize the binding of the other protein contaminants, leaving γ‐conglutin in the supernatant. Finally, the matrix was regenerated with 50 mM Tris‐HCl buffer, pH 7.0, containing 1 M NaCl. For albumin fraction, the same protocol was performed.

### 2.3 Polypeptide and lectin characterization

#### 2.3.1 Polypeptide profile

The discontinuous buffer system described by Laemmli (1970) was used for polyacrylamide gel electrophoresis (PAGE). Two types of electrophoresis were used, namely, nonreducing sodium dodecyl sulfate–PAGE (SDS‐PAGE‐NR) and reducing SDS‐PAGE (SDS‐PAGE‐R), following the methodology previously described (Santos et al., 1997). Before electrophoresis, all protein samples were boiled for 3 min in the presence of sample buffer containing 2% (w/v) SDS (SDS‐PAGE‐NR) or 2% (w/v) SDS and 0.1 M β‐mercaptoethanol (SDS‐PAGE‐R). Gels were stained with silver nitrate (Blum et al., 1987).

#### 2.3.2 Native gels

An electrophoretic run in native conditions was also performed. The resolution gel was composed of 5% (m/v) acrylamide, 2.7% (m/v) bis‐acrylamide, 375 mM Tris‐HCl, pH 8.8, 0.05% (m/v) PSA, and 0,033% (v/v) TEME, and the concentration gel was composed of 3% (m/v) acrylamide, 2,7% (m/v) bis‐acrylamide, 375 mM Tris‐HCl, pH 6.8, 0.05% (m/v) PSA, and 0.1% (v/v) TEME. The samples were treated with sample buffer 4 x concentrated that contained 80 mM Tris‐HCl, pH 6.8, 15% (v/v) glycerol, and 0.01% (v/v) purple m‐cresol. The electrophoretic run was performed in a constant electric current at 20 mA.

#### 2.3.3 Tris‐tricine 16.5% precast gels

The precast gels (Bio‐Rad) were used in the in vitro assays performed with the human insulin. After the electrophoretic run, which was performed following the same methodology described by Santos et al. (Santos et al., 1997), the gel was placed on a different fixing solution in order to fix the proteins with a smaller molecular weight to the gel. The solution was composed of 50% (v/v) methanol, 10% (v/v) acetic acid, and 100 mM ammonium acetate. The gel was placed on this solution for 1 hr, and a silver staining was performed afterward.

#### 2.3.4 Glycoprotein Detection

The glyosidic character of the polypeptide constituents of albumin and globulin fractions was carried out after electrophoretic run (SDS‐PAGE‐R), followed by its transfer and immobilization in nitrocellulose membrane, by the concanavalin A‐Peroxidase method, proposed by Faye & Chrispeels. (1985).

#### 2.3.5 Hemagglutination assays

##### Erythrocyte cells

Rabbit erythrocytes from total blood were treated according to Ribeiro and colleagues (Ribeiro et al., 2012). 5 ml of blood was washed three times in saline and incubated with trypsin for 1h at 37℃ at a final concentration of 1% (v/v). The 4% (v/v) suspension of the trypsinized erythrocytes was stored at 4℃ and used for the hemagglutination activity measurements.

##### Measurements of hemagglutination activity

For hemagglutination activity quantification (Ribeiro et al., 2012), protein extracts (100–250 µg in 50–70 µl saline containing 2 mM CaCl2 and 2 mM MgCl2) were serially diluted (1:3) in a 96‐well microplate. The erythrocyte suspension (50–70 µl) was then added and the microplate incubated for 30 min at 37℃ before visual analysis. Positive (Con‐A lectin at 0.5 mg/ml) and negative (saline) controls were prepared. The hemagglutination unit (H.U.) was defined as the minimal protein concentration, which induces rabbit erythrocyte hemagglutination.

#### 2.3.6 Immunoblotting

##### Production of polyclonal antibodies

Polyclonal antibodies were produced in rabbits against γ‐conglutin from *Lupinus albus*, according to Seabra et al. (1999). Samples containing the purified antigens (200 µg) were mixed with an equal volume of Freund's complete adjuvant (1 ml final volume) and injected subcutaneously into female New Zealand rabbits. To obtain a high titer, three booster injections were given every 2 weeks in complete Freund's adjuvant diluted 1:10 with incomplete adjuvant. After the third booster injection, total blood was taken from the heart 9 days after. Blood samples were allowed to clot, and the serum (with total Ab) was collected and stored frozen at −70℃.

##### Immunodetection

To confirm the purification of γ‐conglutin, an immune detection was performed (Ribeiro et al., 2014). The obtained eluted DEAE matrix was placed on a SDS‐PAGE‐NR, and an electrophoretic run was performed with controls (albumin and globulin fractions) and blotted onto nitrocellulose membranes for immunodetection with polyclonal antibodies prepared against γ‐conglutin.

After protein transfer onto nitrocellulose, the membranes were fixed as described above, then washed for 10 min in PBST (20 mM phosphate buffer, 140 mM NaCl, 20 mM KCl, and 0.05% v/v Tween‐20, pH 7.4) prior to blocking for 1 hr in 1% (w/v) dry skimmed milk in PBST. Specific antibodies (anti‐γ‐conglutin from *lupinus*) were used at 1:750 dilutions in PBST, followed by an incubation of 1 hr at room temperature with gentle shaking. Membranes were washed in PBST twice for 5 min prior to the addition of the secondary antibody specific for rabbit IgGs (A3812; Sigma‐Aldrich), conjugated to alkaline phosphatase, and used at 1:7.000 dilution in PBST. Detection of the immune complex was achieved by hydrolysis of 5‐bromo‐4‐chloro‐3‐indolyl phosphate (BCIP). This reaction was stopped by addition of water. The blot was rinsed twice for 30 s in water and allowed to dry at room temperature.

### 2.4 Structural assays of γ‐conglutin

The purified γ‐conglutin was exposed to a denatured cocktail associated with deglycosylation conditions (mimetic to the release of N‐oligosaccharides). Firstly, γ‐conglutin (45 µg) was mixed with a solution constituted by 2‐mercaptoethanol (2‐Me) 0.1 *M* (v/v) and 0.1% (m/v) SDS, boiled for 3 min, and then placed on ice. In the 2nd step were added in order the following solutions: PBS 100 mM buffer (3 µl), pH 7.2 (2 mM CaCl2 and MgCl2), deionized water (5 µl), 10% (m/v) Triton X‐100 (2 µl), and 250 U/ml of PNGase *F* (3 µl). The mixture was incubated at 37 ºC for 24 hr with slight agitation. The next step was to add a solution of SDS (10×) concentrated (2.5 µl), which contained Tris‐HCl buffer (150 mM Tris‐HCl, pH 6.8 (0.1% SDS), 4% (m/v) SDS, DTT 0.2 M, and 4% (m/v) PBS 100 mM Nonidet P‐40), and the final solution was boiled for 3 min.

### 2.5 γ‐conglutin binding assays to human insulin

Native γ‐conglutin and denatured γ‐conglutin (1,000 µg for each sample in 25 mM Tris‐HCl, pH 7.2, containing 2 mM CaCl2 and 2 mM MgCl2) were incubated with 1,250 µg of human insulin solubilized in the some buffer, with a total reactional volume of about 3 ml. After incubation, in order to remove insulin unbound to native γ‐conglutin and denatured γ‐conglutin, was done one prior centrifugation at 12.096 × g for 10 min, followed by a cycle of three consecutive washes of the sediment, with 15 volumes of saline containing salts, by centrifugation at 12.096 g for 10 min at 4℃ (Beckman J2‐21 641 m/E, Rotor JA 20 000). The supernatant was recovered, and the pellet, consisting of the insulin bound to gamma conglutin, was subsequently solubilized in 0.4 M of NaCl solution, pH 7.2. Aliquots of supernatant and pellet solubilized was measured for protein measurement, to detect whether the insulin bounded to native and denatured γ‐conglutin.

#### 2.5.1 Galactose affinity assays

In parallel was evaluated the effect of galactose 0.4 M (in Tris‐HCL 25 mM, pH 7.2) solution on the dissociation of the pellet formed by γ‐conglutin and insulin, obtained after incubation. In order to clarify this possible effect, the pellet was solubilized in the galactose solution and incubated for 30 min at 25ºC with slight agitation, followed by a centrifugation at 12.096 × *g* for 10 min. If a pellet was visualized, it was solubilized in NaCl 0.4 M, pH 7.2, and the supernatant was passed through a PD‐10 column previously equilibrated in the same solution to eliminate galactose. The solubilized pellet was treated in the same manner to eliminate galactose, which is performed with the hemagglutination assay.

### 2.6 HepG2 cell culture

The HepG2 cells were seeded in plastic flasks and cultured in culture media with 50% of Dulbecco's modified Eagle's medium (DMEM) with low glucose content and 50% of Nutrient Mixture F‐12 Ham and supplemented with 10% (v/v) fetal calf serum, 0.5% (v/v) of penicillin solution at 2 × 104 UI/ml, and 34 mM streptomycin (Oliveira et al., 2019). The cells were kept in a humidified atmosphere containing 5% CO2. The cells were supplied with fresh medium every second day, and trypsinized when the confluence was between 80% and 100%.

#### 2.6.1 Isolation of HepG2 cell membranes

HepG2 cells were grown as described previously, and the membranes were isolated by the method described by Vercoutter‐Edouart et al (Vercoutter‐Edouart et al., 2008). The cells preserved at −80℃ (26 × 10^6^ cells) were thawed rapidly at 37℃, and the suspension of cells was washed with 10 volumes of HES buffer (20 mM HEPES, pH 7.4, and 250 mM sucrose), by centrifugation at 750 g for 10 min, at 21℃ (Beckman J2‐21 m/E, Rotor JA 20,000). The supernatant was discarded, and the pellet was washed twice with HES buffer containing a protease inhibitor cocktail (without EDTA, Roche). Cell lysis was performed by cryolysis, in which cells were subjected serially to freezing and thawing cycles (4 times), during 30 min, at −20℃, and added from a 20‐min sonication in ultrasound, followed by a centrifugation at 960 x *g* for 10 min at 4℃, with discard of the pellet. The final supernatant was ultracentrifuged at 126,000 x *g* for 45 min, 4℃ (Beckman J2‐21 m/E, Rotor SW 32 Ti). The pellet containing the cell membranes was solubilized in 2 ml of physiological saline (0.9% NaCl), containing 2 mM of CaCl_2_ and 2 mM of MgCl_2_, and was divided into aliquots containing 1 mg protein determined by the Bradford method (Bradford, 1976) and kept at ‐ 80℃, until use.

### 2.7 γ‐Conglutin affinity binding to HepG2 cell glycosylated receptors

A protocol according to Oliveira et al. (2019) was followed. The isolated HepG2 cell membranes were individually incubated with the native and the denatured γ‐conglutin. Seven hundred (700 µg) of HepG2 protein membrane was solubilized in 3.5 ml of saline containing 2 mM CaCl_2_ and 2 mM MgCl_2_ and incubated during 45 min at 25℃, by gentle agitation, with 280 µg of native γ‐conglutin and denatured γ‐conglutin, both dissolved in 2 ml of saline containing 2 mM CaCl2 and 2 mM MgCl2. After incubation, in order to remove the protein unbound to membranes, was done one prior centrifugation at 12.096 g for 10 min, followed by a cycle of three consecutive washes of the pellet with 15 volumes of saline containing salts and centrifugation at 12.096 x *g* for 10 min at 4℃ (Beckman 684 J2‐21 m/E, Rotor JA 20,000). The supernatant was discarded, and the obtained pellet, consisting of a complex formed between protein and membranes, was subsequently solubilized in saline solution (containing salts) and used, after protein measurement, to detect whether the native and denatured γ‐conglutins were bounded to the membranes. The control was made by using saline instead of native and denatured γ‐conglutins in the membrane incubation. The complex binding was visualized by 1D electrophoresis.

#### 2.7.1 Proteomic profile of HepG2 Cell membranes by 2D analysis

Two‐dimensional (2D) electrophoresis (IEF/SDS‐PAGE‐R) of the cell membranes of HepG2 was carried out as follows, according to Oliveira et al. (2019). It was applied in the first dimension, isoelectric focusing (IEF), 1,400 µg of HepG2 membrane protein, separated using the IPGphor system (Amersham Pharmacia).

The second dimension (SDS‐PAGE‐R) was performed after IEF. The gel strips were thawed and equilibrated for 15 min, with stirring, in 50 mM Tris‐HCl buffer, pH 8.8, containing 6 M urea, 30% (v/v) glycerol, 2% (w/v) SDS, and 1% (w/v) dithiothreitol. The strips were subsequently equilibrated for another 15 min, with agitation, in a similar solution that contained 2.5% (w/v) iodoacetamide instead of dithiothreitol, placed on top of a 17.5% (w/v) acrylamide SDS‐PAGE gel, sealed with 0.7% (w/v) agarose (containing 0.002% (w/v) bromophenol blue), and electrophoresed (220 V, 15 mA for 15 min followed by 220 V, 30 mA). After electrophoresis, the gels were stained by silver nitrate (Blum et al., 1987).

### 2.8 In vivo assays

#### 2.8.1 Animals

Male CD‐1 mice with 30–40 g body weight and 6–10 weeks old (Instituto de Higiene e Medicina Tropical) were used in the hyperglycemic study.

Animals were accommodated in polypropylene cages with free access to water and food and kept at a temperature of 18–23ºC and humidity of 40%–60% in a 12‐hr light/dark cycle at the Animal Facility of the Faculty of Pharmacy (University of Lisbon). Experiments were performed agreeing to the most recent rules and recommendations for the care and processing of laboratory animals, namely to the presently adopted European Commission regulations (Directive 2010/63/EU).

In addition, the studies were performed in agreement with the ARRIVE Guidelines for Reporting Animal Research. All animal experiments were conducted according to the animal welfare organ of the Faculty of Pharmacy, Universidade de Lisboa (project 0019/2018 submitted and approved), in representation of the competent national authority Direção‐Geral de Alimentação e Veterinária (DGAV) and in accordance with the EU Directive (2010/63/UE) and Portuguese laws (DR 113/2013, 2880/2015, and 260/2016).

#### 2.8.2 Administration

Animals were administered daily by gastric gavage during 7 days with the last administration being at the final day, 30 min prior to basal glycemic measurement and hyperglycemic induction.

Animals were randomly allocated to five experimental groups:

Normoglycemic group—animals were treated orally with vehicle for 7 days, and at the last day, water was administered instead of glucose;

Hyperglycemic group—animals were treated with vehicle for 7 days, and at the last day, a solution of glucose (65 mg/kg) was administered by gastric gavage;

Native γ‐conglutin group—animals were treated orally with γ‐conglutin (60 mg/kg BW) for 7 days, and at the last day, a solution of glucose (65 mg/kg) was administered by gastric gavage;

Denatured γ‐conglutin group—animals were treated orally with denatured γ‐conglutin (60 mg/Kg BW) for 7 days, and at the last day, a solution of glucose (65 mg/kg) was administered by gastric gavage;

Metformin group—animals were treated orally with metformin (300 mg/kg BW) for 7 days, and at the last day, a solution of glucose (65 mg/kg) was administered by gastric gavage.

##### Experimental hyperglycemia

A basal measurement was performed at t0 immediately before administration of the glucose solution, and subsequent measurements of glycemia were performed at t30, t60, and t120 after glucose administration. Glycemic measurements were performed by collection of a blood drop by means of tail vein puncture and determined with a portable glucometer.

##### Blood collection

At the end of the experiment, animals were anesthetized by administration of a mixture of ketamine:xylazine (80 mg/kg:8 mg/kg) and blood was collected by cardiac puncture for measurement of pancreatic (insulin, amylase, and lipase), renal (creatinine and urea), hepatic (alanine aminotransferase and aspartate aminotransferase), and lipid (total cholesterol, HDL‐cholesterol, LDL‐cholesterol, and triglycerides) serum biomarkers. All these markers were evaluated in a clinical analysis laboratory certified under the ISO9001‐2015 standard. The analytical determination was made on an autoanalyzer COBAS C702™ (Roche Diagnostics GmbH) following the manufacturer's instructions (Roche Diagnostics GmbH) for the determination of each biomarker. LDL‐cholesterol is measured with an homogeneous enzymatic colorimetric assay developed by Roche Diagnostics GmbH. Briefly, cholesterol esters and free cholesterol in LDL are measured on the basis of a cholesterol enzymatic method using cholesterol esterase and cholesterol oxidase in the presence of surfactants, which selectively solubilize only LDL. The enzyme reactions to the lipoproteins other than LDL are inhibited by surfactants and a sugar compound. Cholesterol in HDL, VLDL, and chylomicron is not determined by this method. Animals were subsequently euthanized by anesthetic overdose.

### 2.9 General assays

Protein quantification was made by a modification of the Bradford method (Bradford, 1976), with bovine serum albumin used as the standard. SDS‐PAGE, PAGE native, and 2D gels were stained by CBB R‐250, CBB G‐250 (Neuhoff et al., 1988), or silver stained (Blum et al., 1987).

### 2.10 Statistical analysis

All results were expressed as mean ± S.E.M. of n observations, where n represents the number of animals studied. Results were compared using a two‐factorial ANOVA test, followed by a Bonferroni's post hoc test using GraphPad Prism 5.0 software (GraphPad). A *p* value less than 0.05 was considered to be statistically significant.

## 3 RESULTS

### 3.1 γ‐Conglutin characterization

**FIGURE 1:**
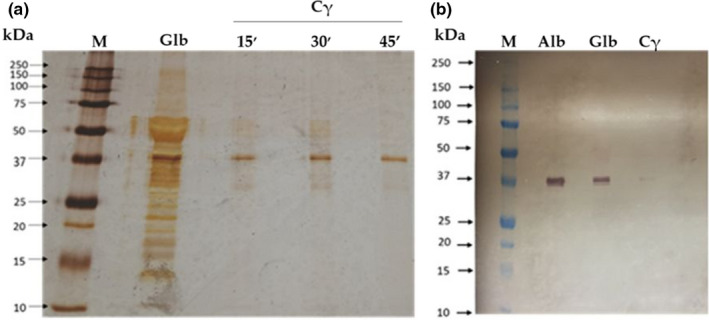
γ‐Conglutin purification (a) Supernatants containing γ‐conglutin (Cγ) from DEAE‐Sepharose, after different contact times with matrix and globulin fraction. Polypeptide profile by a SDS‐PAGE‐NR, 17.5% (m/v) acrylamide revealed by silver staining. It was applied 7 µg of total globulin fraction (Glb) as control and γ‐conglutin (Cγ) and 3 µg of molecular markers (M). (b) Immunodetection in a nitrocellulose membrane using anti‐ γ‐cong. (lupinus) as primary antibody and anti‐rabbit IgG as secondary antibody. It was applied 15 µg of globulins (Glb) and albumins (Alb) as control, 10 µg of purified γ‐conglutin supernatant (Cγ), and 12 µl of molecular weight (M)

γ‐Conglutin was purified from the globulin fraction of *Lupinus albus* seeds by DEAE‐Sepharose under optimized batch conditions during 45 min at 25℃, and stirred every 15 min, as revealed by SDS‐PAGE performed under nonreducing conditions (Figure 1a). Previously, both total albumin and total globulin fractions were assayed as potential sources for γ‐conglutin purification. However, the globulin fraction results in a more efficient γ‐conglutin purification. A molecular weight of γ‐conglutin of about 37 kDa (Figure 1b) was confirmed by immunodetection using a polyclonal antibody against *Lupinus albus* γ‐conglutin, a value which is in good agreement with that described as 42 kDa in the available literature.

In order to confirm γ‐conglutin lectin character, the hemagglutination activity was measured by a hemagglutination assay. For amounts of protein tested 15 and 30 µg, a hemagglutination unit (H.U.) of 0.14 µg ± 0.05 µg was obtained for purified γ‐conglutin isolated from the globulin fraction, signifying that this value is the minimal protein concentration that produces erythrocyte hemagglutination. The controls used were saline as the negative control and concanavalin A (500 µg/ml) as the positive control.

### 3.2 γ‐Conglutin denaturation

**FIGURE 2:**
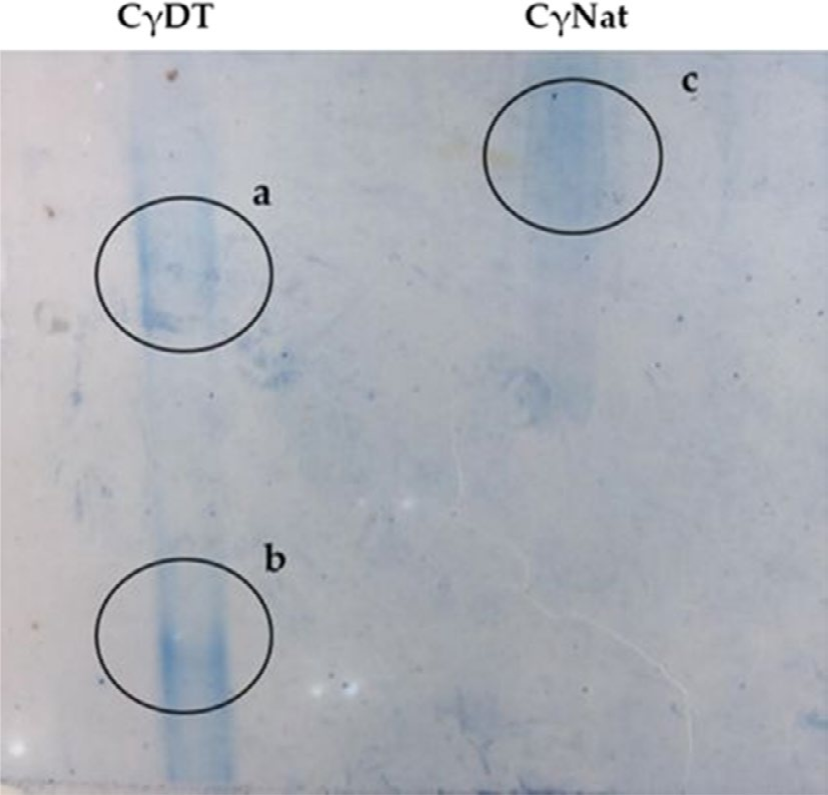
γ‐Conglutin denaturation. Native PAGE and 5% (m/v) acrylamide revealed by Coomassie G. It was applied 20 µg of denatured γ‐cong. (CγDT) and native γ‐cong. (CγNat)

In order to denature γ‐conglutin, the purified protein was exposed to a denaturing “cocktail” associated with deglycosylation conditions. In an attempt to understand the consequences of incubating γ‐conglutin with this “cocktail,” an electrophoretic analysis under native conditions was performed, with native γ‐conglutin as the control. Figure 2 reveals the presence of two polypeptide bands in the denatured form, highlighted by circles (a) and (b), and one protein band in the native form of γ‐conglutin (c), which confirmed the denaturation of γ‐conglutin.

### 3.3 Molecular binding skills of native and denatured γ‐Conglutins

#### 3.3.1 Binding capacities to human insulin

**FIGURE 3:**
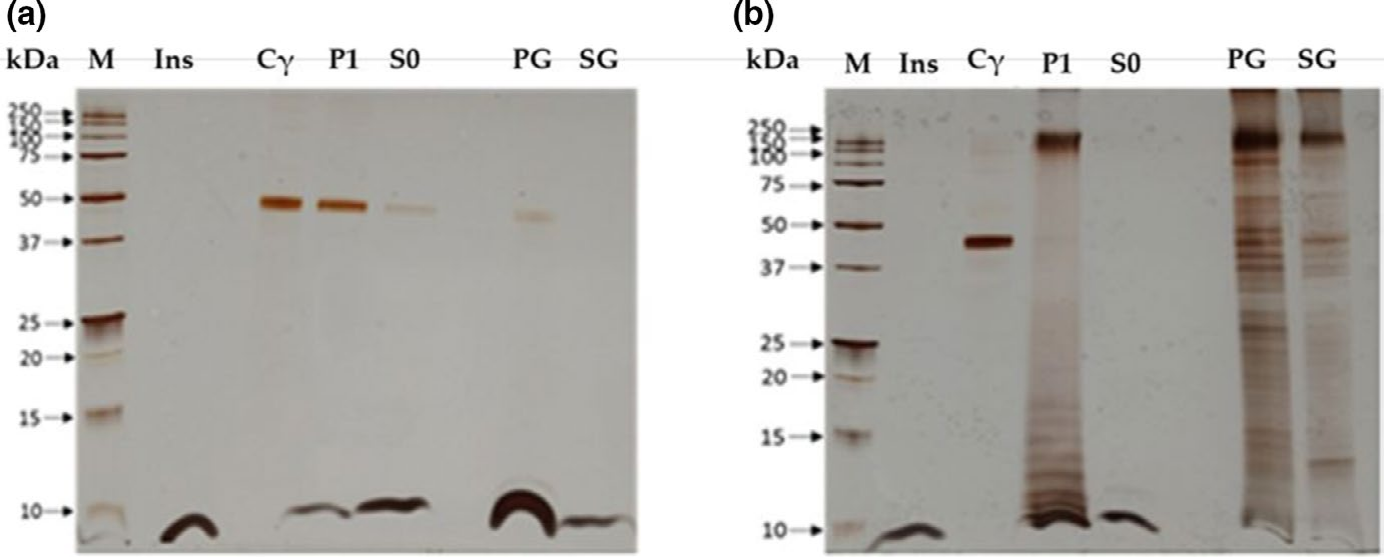
Binding of native and denatured γ‐conglutin forms to human insulin. (a) Binding assay of native γ‐conglutin with human insulin. (b) Binding assay of denatured γ‐conglutin with human insulin. A gel Tris‐Tricine, 16.5% (m/v) in acrylamide, staining by silver nitrate was performed. It was applied 3 µl of molecular markers (M), 6 µg of both human insulin (Ins) and γ‐conglutin as control, and 13 µg of pellet (P1) and supernatant (S0) and 26 µg of galactose pellet (PG) and galactose supernatant (SG)

To study the binding capacities to human insulin, both native and denatured γ‐conglutins were incubated with insulin, having ensured previously the solubilization of all molecules (Materials and Methods section). For each γ‐conglutin (native with lectin activity and denatured), two incubations were performed with insulin and the resulting pellet was collected for each one. The first incubation of native γ‐conglutin incubation with insulin results in a pellet, which was washed in Tris buffer and solubilized in NaCl 0.4 M, pH 7.2 (Figure 3a (P1)), and in a supernatant (Figure 3a (S0). The second incubation results in a pellet of the denatured γ‐conglutin incubation with insulin (Figure 3b (P1)) and a supernatant (Figure 3b (S0). The first one, in a second step, was incubated with galactose 0.4 M (Figure 3b (PG)), resulting a supernatant (Figure 3b (SG). In both assays, controls of native γ‐conglutin (Cγ) and human insulin (Ins), both under nonreducing conditions, were applied (Figure 3). Analyzing Figure 3 and considering P1 (incubation pellet solubilized in 0.4 M NaCl, pH 7.2), S0 (supernatant that represents protein not binding to insulin), PG (pellet obtained in the galactose assay, solubilized in 0.4 M NaCl, pH 7.2), and SG (supernatant from galactose assay), evidenced by Tris‐Tricine SDS‐PAGE‐NR and 16.5% (w/v) acrylamide gel, the following results were inferred: In Figure 3a, considering controls, it was revealed that native γ‐conglutin binds to human insulin (P1) and the unbound protein (S0) is mainly represented by insulin, meaning that almost all γ‐conglutin was attached to insulin, and when the pellet was solubilized with galactose 0.4 M, a glycan for which γ‐conglutin exhibits a preferential binding specificity, the pellet did not dissociate, thus maintaining the preference of γ‐conglutin binding to insulin, with the appearance of two polypeptide bands of 42 kDa and 6 kDa, respectively γ‐conglutin and insulin (PG), leaving the unbound insulin in the supernatant (SG). Concerning the experiments on denatured γ‐conglutin binding to human insulin (Figure 3b), the results of the incubation evidence a complex polypeptide profile, when compared to native γ‐conglutin control, represented principally by polypeptide bands of low molecular weight of 10 to 20 kDa, and others with differenced molecular weights, among which is noted a major oligomeric polypeptide band about 150 kDa, as result of the severe treatment suffered by the sample subjected to the denaturation applied, but retaining its binding capacity to human insulin (Figure 3b(P1)), with a clear visualization of the 6 kDa insulin protein band. The supernatant (S0) shows the excess of human insulin that did not bind to denatured γ‐conglutin. The pellet incubated with galactose (PG) revealed a polypeptide profile of denatured γ‐conglutin that exhibit a vestigial mark of insulin, at molecular mass level of 6 kDa, meaning that insulin was partially or totally detached from denatured γ‐conglutin by galactose. Also in the supernatant (SG), insulin is not clearly visible, raising doubts about the fate of insulin after incubation with galactose 0.4 M.

### 3.4 Binding of γ‐Conglutin to human hepatocyte cell membranes (HepG2)

#### 3.4.1 Polypeptide profile characterization of HepG2 cell membranes

**FIGURE 4:**
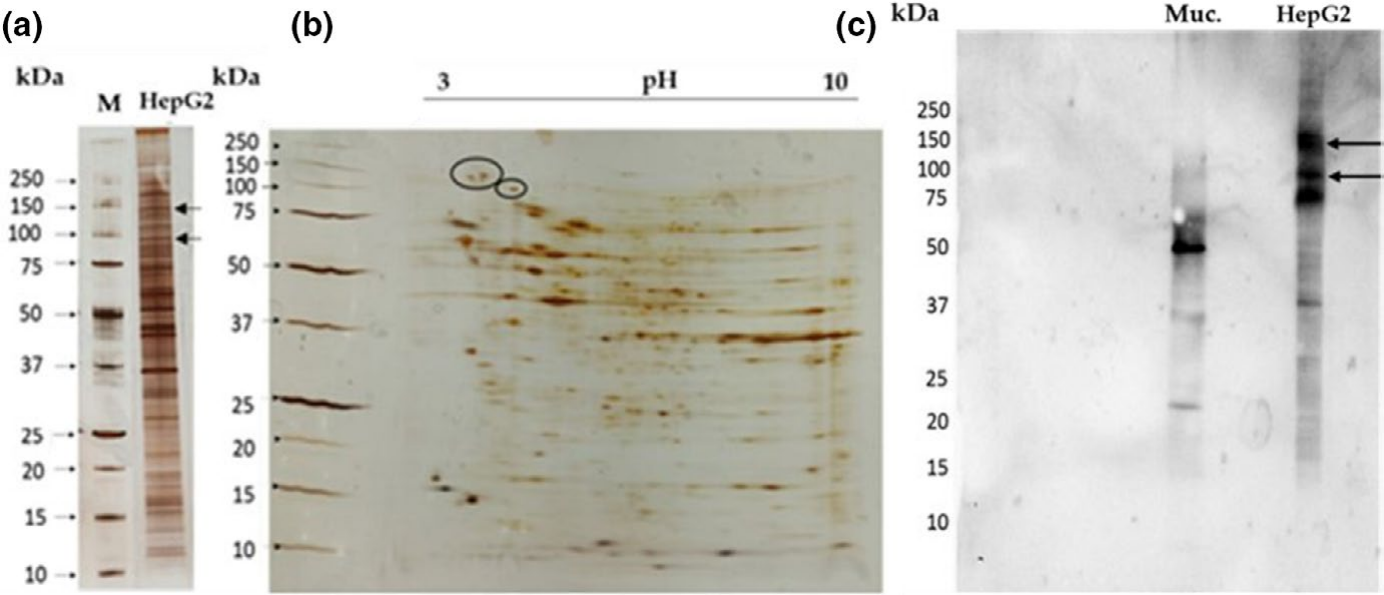
Proteomic profile of HepG2 cell membrane. (a) Polypeptide profile of HepG2 cells in a SDS‐PAGE‐R, with 17.5% (m/v) of acrylamide. It was applied 3 µg of molecular marker (M) and 13 µg of HepG2 cell membranes. (b) Two‐dimensional analyses of HepG2 cell membranes (IEF/SDS‐ PAGE‐R), in a gel SDS‐PAGE‐R, with 17.5% in acrylamide. It was applied 1,300 µg of HepG2 cell membranes and 5 µl of molecular markers (M). The ellipse identified the insulin receptors. (c) Glycodetection on HepG2 cell membranes in a nitrocellulose membrane. The arrows refer to insulin receptors. It was applied 50 µg of mucin (Muc) and 52 µg of HepG2 membranes

Polypeptide characterization analysis of HepG2 cell membranes by one‐dimensional (SDS‐PAGE‐R) and two‐dimensional (IEF/SDS‐PAGE‐R) electrophoreses was performed. Results of the SDS‐PAGE‐R (Figure 4a) showed a complex polypeptide profile, covering an extended molecular weight range (between 10 and 200 kDa) with several prominent representative bands (range 30 kDa to 75 kDa), some of which with a particular interest such as the insulin receptors of 90 kDa and 135 kDa already described.

The 2D electrophoresis (Figure 4b) revealed a profile with many polypeptide spots, with pI values ranging between pH 3 and pH 10. Higher molecular weights (50 to 150 kDa) include the insulin receptors with two isoforms, ca. 130 kDa with pI around 3.9 and 4.1., and one other with a molecular weight of 95 kDa and pI around 4.7. These are highly represented spots and are spread in the acidic zone (pH 3 to 6). For lower molecular weights (30 to 37 kDa), several well‐represented polypeptides are spread in the basic zone (pH 8 to 10). In the neutral zone, diverse spots with different molecular weights are visible, but, in these cases, with less representativeness.

As the glycomic profile is very important to characterize lectin glycoreceptors, the study of the glycoprotein content in the HepG2 cell membranes was done. The purified HepG2 cell membrane polypeptides were separated by SDS‐PAGE‐R, which was used to transfer the polypeptide profile of these membranes to a nitrocellulose membrane. In this assay, the positive control used was mucin, a well‐known glycoprotein. As shown in Figure 4c, the HepG2 cell membrane polypeptide profile is poor in glycoproteins. Only a few glycosylated bands were revealed, and the most representative ones exhibit high molecular weights. The insulin receptors are marked in Figure 4c with an arrow highlighting the polypeptide bands of 95 and 130 kDa.

#### 3.4.2 Binding of native and denatured γ‐conglutin to HepG2 membrane receptors

**FIGURE 5:**
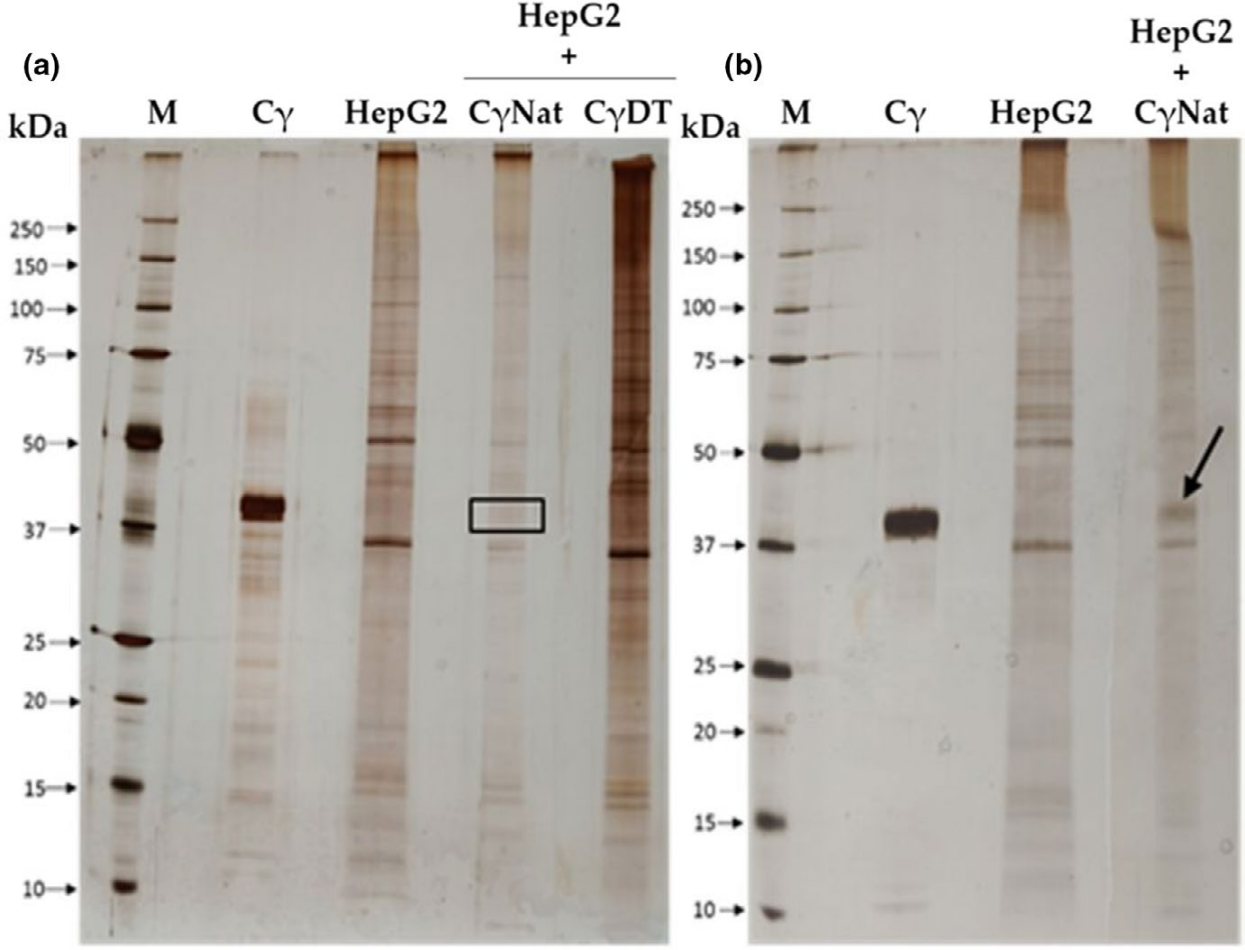
Binding of denatured and native γ‐conglutin to membrane receptors of HepG2 cells: A SDS‐PAGE‐NR and 17.5% in acrylamide (m/v) revealed by AgNO3 staining was performed. (a) M—molecular mass markers; Cγ—native γ‐conglutin (control); HepG2—cell membranes (control); HepG2+ CγNat—incubation of membranes with native γ‐conglutin without lectin activity; HepG2+CγDT—incubation of membranes with denatured γ‐conglutin. (b) M—molecular mass markers; Cγ—native γ‐conglutin (control); HepG2—cell membranes (control); Native γ‐conglutin (control); HepG2 membranes control; HepG2+ CγNat—incubation of membranes with native y‐conglutin with lectin activity. It was applied 3 µg of molecular mass markers, 6µg CγNat, and 13 µg of HepG2, HepG2+ CγNat with and without activity and HepG2+CγDenatured. The arrow highlights γ‐conglutin polypeptide bound to HepG2 cell receptors

The study of the lectin character on the binding capacity of γ‐conglutin (with and without lectin activity) to HepG2 cells was evaluated. An incubation of HepG2 cell membranes with three forms of γ‐conglutin was analyzed: native γ‐conglutin without lectin activity (batch 1), native γ‐conglutin with lectin activity (batch 2), and denatured γ‐conglutin (see Table 1). The protein profiles were analyzed using purified HepG2 cell membranes and native γ‐conglutin as controls. Figure 5a shows that after incubation of HepG2 cell membranes with native γ‐conglutin without lectin activity and denatured γ‐conglutin, the γ‐conglutin in the absence of lectin activity did not bind to the cell membrane receptors, since the polypeptide profile of the membrane control is identical to the polypeptide profile of the resulting membrane incubation pellet. On the other hand, the incubation of native γ‐conglutin with lectin activity with HepG2 cells (Figure 5b) revealed a link between this protein and the glycan receptors of HepG2 cell membranes, since a polypeptide band matched with γ‐conglutin control with a molecular weight near 42 kDa is evident in the HepG2 protein profile (highlighted by an arrow).

**TABLE 1:** Hemagglutination activity detection in rabbit erythrocytes

Protein fraction	Hemagglutination activity (H.U)(µg)
Native γ‐conglutin batch 1	ND
Native γ‐conglutin batch 2	4.35
Denatured γ‐conglutin	ND

Abbreviations: H.U, minimal protein concentration that gives hemagglutination; ND, not detected

### 3.5 In vivo evaluation of the hypoglycemic effect of γ‐Conglutin

**FIGURE 6:**
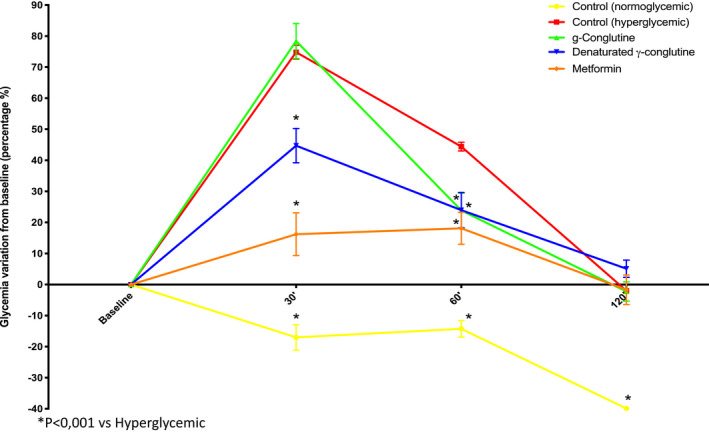
In vivo evaluation of the hypoglycemic effect of native γ‐conglutin and denatured γ‐conglutin. Five mouse groups were tested: hyperglycemic group control, normoglycemic group control, group exposure to native γ‐conglutin, group exposure to denatured γ‐conglutin, and group exposure to metformin. It was tested 60 mg/kg BW CD‐1 mouse of the native γ‐conglutin and denatured γ‐conglutin and 300 mg/kg BW for the metformin group

The antihyperglycemic effect of γ‐conglutin was evaluated in male mice (CD‐1) by an in vivo protocol described in the Materials and Methods section. The data showed that native γ‐conglutin (Cγ‐Nat) exhibited an antihyperglycemic effect (Figure 6). After the first 30‐min postglucose administration, the level of serum glucose rise is approximately 80%, similar to the values obtained for the hyperglycemic control group, and 60% higher than that of the metformin (a well‐known drug used in the treatment of type 2 diabetes) values. After 60 min, the level of blood glucose decreases approximately 50% in the Cγ‐Nat group, and the relative levels of glycemic reduction at this time point are comparable to the results obtained for the group treated with metformin. After 120 min, there is an intersection of blood glucose levels among the hyperglycemic control group, the group treated with the native γ‐conglutin, and the group treated with the metformin, all with serum glucose relative levels of 0%, meaning that the glucose levels in the blood returned to basal values after the 120 min for all of these groups.

For the group treated with denatured γ‐conglutin (Cγ‐DT), the antihyperglycemic effect is visible during the first 30 min of this assay (Figure 6). At this time, the glucose levels in the blood increase approximately 45%, which is almost half of the rise obtained for the group treated with native Cγ and the hyperglycemic control. In the group treated with metformin, the glucose levels in the blood raised approximately 15% after the first 30 min. The results obtained for the group treated with Cγ‐DT are closer to the group treated with metformin in comparison with the group treated with the native γ‐conglutin (Cγ‐Nat). The glucose levels in the blood continued to decrease at 60 min, with relative glucose values similar to the metformin group. At this time, there is a line intersection between for the two groups treated with the different forms of γ‐conglutin studied. The glucose levels in the blood of the Cγ‐DT group are the same as in both groups mentioned, and there is not a significant difference between these groups and the one treated with metformin, which evidences the antihyperglycemic effect of the native and denatured γ‐conglutin. Finally, after 120 min the glucose levels continue to decrease to basal values. At this time, there is no significant difference between the groups treated with denatured or native γ‐conglutin and the one treated with metformin.

#### 3.5.1 Exposure evaluation to γ‐conglutin in the expression of organ injury/functional biomarkers

##### Pancreatic functional biomarkers

**FIGURE 7:**
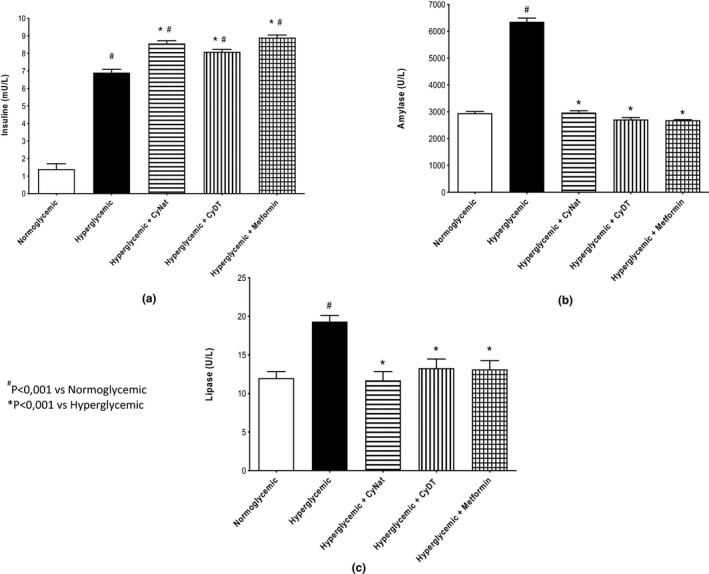
Pancreatic functional biomarkers—Evaluation of the exposure of native (Cγ‐Nat) and denatured γ‐conglutin (Cγ‐DT) in the expression of insulin (a), amylase (b), and lipase (c) and comparison with normoglycemic group, hyperglycemic group, and metformin exposure group

The results obtained for pancreatic biomarkers when γ‐conglutin is administered to mice are evidenced in Figure 7. The biomarkers studied were insulin, amylase, and lipase (Figure 7a,b,c, respectively). In comparison with the normoglycemic group (control), all the other groups (treated with native and denatured γ‐conglutin, the hyperglycemic control group, and the one treated with metformin) showed increased values of insulin in the blood with no significant difference in insulin values among these groups albeit slightly lower for the glucose group (hyperglycemic group).

Amylase only increased in the hyperglycemic group control. The groups treated with the native and denatured γ‐conglutin and the metformin group revealed no significant difference when compared to the normoglycemic group control. The same scenario happened with lipase. This enzyme only increases in the hyperglycemic group control. All the other groups presented similar values of this enzyme, and there is no significant difference among them.

##### Liver injury biomarkers

**FIGURE 8:**
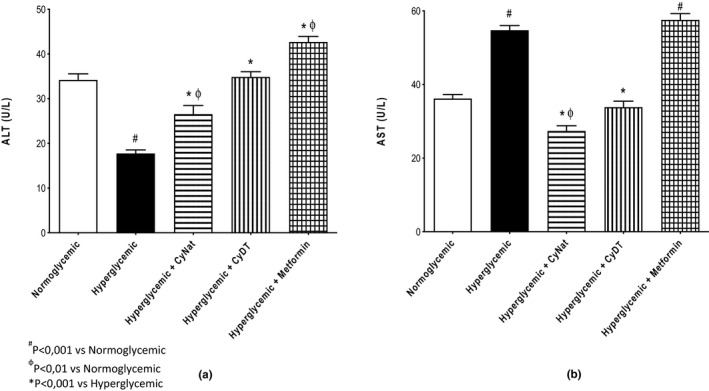
Liver function biomarkers—Evaluation of the exposure of native (Cγ‐Nat) and denatured γ‐conglutin (Cγ‐DT) in the expression of ALT (a) and AST (b) and comparison with normoglycemic group, hyperglycemic group, and metformin exposure group

The results for the liver biomarkers when γ‐conglutin is administered to mice are shown in Figure 8. The biomarkers evaluated were alanine aminotransferase (ALT) (Figure 8a) and aspartate aminotransferase (AST) (Figure 8b). For the ALT, the results showed that the Cγ‐DT group presented values similar to the normoglycemic control group while the Cγ‐Nat group exhibited 10% lower values and metformin group 10% higher values.

The AST value obtained for the groups treated with native (Cγ‐Nat) and denatured γ‐conglutin (CyDT) was lower than that of the normoglycemic group control, although not significantly, and much lower than the group treated with metformin, about 50%, and 40% lower, respectively. The hyperglycemic control group and the metformin group revealed similar AST levels, being the highest among all groups.

##### Renal functional biomarkers

**FIGURE 9:**
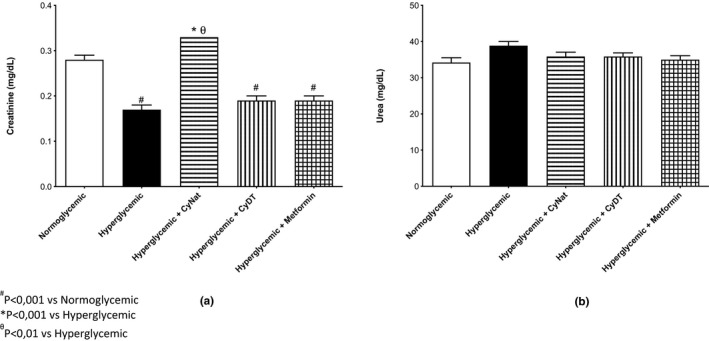
Renal function biomarkers biomarkers—Evaluation of the exposure of native (Cγ‐Nat) and denatured γ‐conglutin (Cγ‐DT) in the expression of creatinine (a) and urea (b) and comparison with normoglycemic group, hyperglycemic group, and metformin exposure group

The renal functional biomarkers evaluated in this assay were creatinine and urea, and the results are displayed in Figure 9a,b. Regarding creatinine, the groups treated with denatured γ‐conglutin and metformin and the hyperglycemic group control revealed very similar values between them, significantly lower than the normoglycemic group. The group treated with native γ‐conglutin (Cγ‐Nat) is the group with the highest value of creatinine, although with no significant difference when compared to the normoglycemic group control. Regarding urea, all the groups in the study revealed similar values, and there are no significant differences between them.

##### Lipid profile biomarkers

**FIGURE 10:**
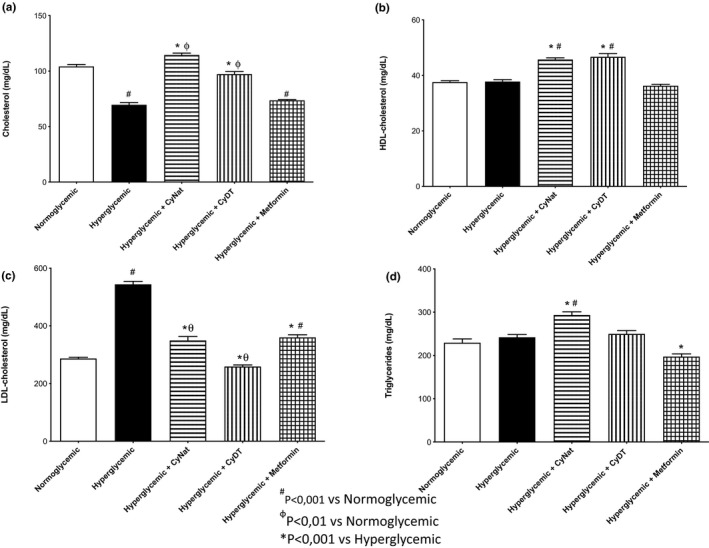
Dyslipidemia biomarkers—Evaluation of the exposure of native (Cγ‐Nat) and denatured γ‐conglutin (Cγ‐DT) in the expression of cholesterol (a), HDL (b), LDL (c), and TG (d) and comparison with normoglycemic group, hyperglycemic group, and metformin exposure group

The lipid profile biomarkers studied were total cholesterol, HDL, LDL, and triglycerides (TG), and the results for all these biomarkers are exhibited in Figure 10a,b,c,d, respectively. The results for total cholesterol levels revealed that this biomarker value for the normoglycemic group control is similar to the groups treated with the native and denatured γ‐conglutin (slightly lower). The metformin group and the hyperglycemic group control showed very similar values of the total cholesterol content. Regarding β‐ME (Figure 10b), the results showed that, for this biomarker, the same values were obtained for the normoglycemic and hyperglycemic group controls and also for the group treated with metformin. The groups treated with denatured and native γ‐conglutin revealed higher values of HDL, but the difference between these values is not significant in comparison with the other groups evaluated.

The group with the lowest LDL levels is the group treated with denatured γ‐conglutin (230 mg/dl), although this result is not significantly different than the one obtained for the normoglycemic group control, having increased when compared to the hyperglycemic group. The groups treated with native γ‐conglutin (Cγ‐Nat) and metformin exhibited very similar values of LDL. This value is lower than that obtained for the hyperglycemic group control and higher than that obtained for the normoglycemic group control (300 mg/dl). The hyperglycemic group control has the highest value (550 mg/dl) of LDL values.

Regarding TG, results revealed no difference between normoglycemic, hyperglycemic, and CyDT group values (≈230 mg/dl). The group treated with native (Cγ‐Nat) exhibited values higher (≈290 mg/dl) than the control groups, but the difference between these is not significant. The group treated with metformin revealed the lowest value of TG content (≈200 mg/dl).

## 4 DISCUSSION

Diabetes is a chronic disease that affects nearly 420 million of people in all world (Laemmli, 1970), being one of the leading causes of death (World Health organization). Type 2 diabetes mellitus (T2D) is characterized by insulin resistance and the inability of the β cell to compensate for it (Zatterale et al., 2020).

In the last years, plant‐based research has been improving via increased ethnobotanical and ethnopharmacological knowledge, more precisely in legume seeds consumed on a daily basis. This allowed for the establishment of a relationship between chronic intake and exhibition of nutraceutical and functional behavior, which resulted in health benefits (Arnoldi et al., 2015; Guzmán et al., 2019; Scarafoni et al., 2007). *Lupinus* is a leguminous plant whose seeds have been consumed as a functional food and revealed pharmacologic activity with hypoglycemic effect, evidenced by different metabolic mechanisms, such as enhanced glucose reuptake and reduction in gluconeogenesis in vitro (Magni et al., 2004; Muñoz et al., 2018). This is of importance as it can be used in the managements of diabetes through modulation of different targets (insulin, glycosylate insulin receptors, and glucose transporters). Studies with animals and humans indicate that moderate consumption of seeds of the genus *Lupinus* or their derived and toxic alkaloids has positive effects on hyperglycemia and glucose homeostasis (Wink, 2005.

γ‐Conglutin is a seed protein exclusive to seeds of *Lupinus* species with documented pharmacologic activity regarding the hypoglycemic activity in both in vitro and in vivo models and human studies.

The first aim of this work was to understand the influence of the lectin character of γ‐conglutin on its hypoglycemic activity. Until now, no study has been reported based on this property in order to understand whether γ‐conglutin hypoglycemic activity remains effective when its lectin activity is absent. This fact is highly relevant as it can influence the behavior of γ‐conglutin binding to target molecules such as insulin, membrane insulin receptors, or glucose transporters, among others. Native γ‐conglutin may have specificity for glycan components in these target molecules. Insulin is the only molecule among the others studied in this work that is not glycosylated, meaning that γ‐conglutin lectin activity is not directly involved in insulin binding.

The binding studies carried out between human insulin (MW 6–10 kDa) and: (a) purified native γ‐conglutin with lectin activity (H.U. = 0.14 ± 0.05 µg); (b) γ‐conglutin without lectin activity (Table 1), and (c) denatured γ‐conglutin, which revealed that the presence of lectin activity is not required for γ‐conglutin binding to insulin (Figure 3a‐P1), as also its native structure is not demanding (Figure 3b‐P1), meaning that the insulin binding capacity of γ‐conglutin is not lectin‐mediated. The solubilization of the resulting incubation pellets (Figure 3a‐P1 and Figure 3b‐P1) with 0.4 M NaCl, followed by incubation with 0.4 M galactose, do not move γ‐conglutin from the complex formed with insulin as γ‐conglutin binds strongly to insulin. However, in the case of denatured γ‐conglutin, this interpretation is dubious because the polypeptide profile after incubation in the presence of galactose seems to reveal trace amounts of insulin (Figure 3b‐PG and SG) suggesting the rupture of the complex. From Figure 3b, it is observed that the aggregates in denatured γ‐conglutin (Figure 3b‐P1 and PG) could be due to the structural pH changes where the incubation medium has a pH near 7.5 resulting in oligomeric forms of γ‐conglutin of 100, 250, and 480 kDa, as also hexameric forms (Capraro et al., 2010).

It has been described that γ‐conglutin binds to insulin via electrostatic interactions, by an unknown mechanism, which required its native conformation to established this bound (Magni et al., 2004). The present results added interpretation, as neither NaCl 0.4 M, nor galactose 0.4 M, can destroy the bonds between native γ‐conglutin and human insulin (Figure 3a‐PG), in a concentration enough for to do the rupture of the most electrostatic forces, revealing that strong electrostatic forces are present.

The binding activity of denatured γ‐conglutin to insulin is new, adding new interpretations to the behavior of γ‐conglutin. These results are supported by an in vitro study (Muñoz et al., 2018where γ‐conglutin hydrolysates from Andean lupin (*L*. *mutabilis*) were assayed. The authors reported a hypoglycemic activity of γ‐conglutin hydrolysates suggesting inhibition of dipeptidyl peptidase IV activity (DPP‐IV) (100%) and an increase of 6.5‐fold in the glucose uptake in a dual‐layered enterocyte/adipocyte culture system compared with untreated cells, resulting in an enhancement of glucose transporter type 4 translocation with insulin‐induced glucose uptake potentiated by the addition of *L*. *mutabilis* hydrolysates and a decrease of 50% gluconeogenesis. By analogy, the present work adds information about insulin binding by denatured γ‐conglutin that, along with the results from the in vivo assays, evidences a bioactivity after 60 min of administration, resulting in lower glycemic levels (Figure 6).

Concerning the insulin receptor (INS‐1) expressed in cell membranes, it has a MW of 300 kDa and is formed by two polypeptide chains of 130 and 95 kDa (Lawrence et al., 2007; Meyts, 2008). The heavily glycosylated human insulin receptor has two glycosylation types, N‐glycosylation and O‐linked glycans (type Gal‐NAcGal‐NeuAc or GalNAc‐Gal expressed in CHO‐K1 cells). The O‐glycans are attached to six of the nine S/T residues in the N‐terminal tryptic peptide of insulin β‐chain (Sparrow et al., 2007), suggesting that γ‐conglutin may show specificity to these domains as it is galactose‐specific (Ribeiro et al., 2014). Others authors (Klein et al., 2009) reported that the accumulation of INS‐1 Ser1011 GlcNAc in HepG2 liver cells and MC3T3‐E1 preosteoblasts upon inhibition of O‐GlcNAcase indicates that O‐GlcNAcylation of endogenously expressed INS‐1 is a dynamic process that occurs at normal glucose concentrations (5 mM).

In this work, HepG2 cell membranes purified by an optimized methodology (Oliveira et al., 2019; Vercoutter‐Edouart et al., 2008) were analyzed by 1D and 2D (IEF/SDS‐PAGE‐R) electrophoresis for proteomic profiling (Figure 4a,b) analysis, followed by glycodetection of polypeptide profile of these membranes(Figure 4c), for INS‐1 detection as identified in Figure 4a,b,c. The glycoprotein profile of HepG2 cell membranes revealed a relatively simple pattern comprising a few glycoproteins, with the INS‐1 receptors localized at the MW described of 130 kDa, for the heavy polypeptide chain, when compared to mucin control of 50 KDa, with two isoforms with pI around 3.8 and 4.1, and of 95 kDa for the lower polypeptide chain with pI near pH 4.5 (Figure 4b). When the incubation of the cell membranes was performed with: (a) γ‐conglutin lacking lectin activity (CϒNat+HepG2) and (b) denatured γ‐conglutin (CϒDT+HepG2) (Figure 5a), the results obtained for polypeptide profile, taking into account the controls of HepG2 cell membranes (HepG2) and γ‐conglutin (Cy), revealed that the lectin activity and native conformation of γ‐conglutin are mandatory for its binding to glycosylated membrane receptors (HepG2 +CϒNat) (Figure 5b). When lectin activity of γ‐conglutin is absent and when γ‐conglutin is denatured, no γ‐conglutin‐glycosylated membrane receptor binding was detected (Figure 5a). These findings suggest new concepts in the interpretation of antihyperglycemic action mediated by insulin receptors, exerted by γ‐conglutin in cell membrane extrinsic binding, suggesting that it is viable only when native γ‐conglutin exhibits lectin activity. Considering these results it becomes truly important to clarify the antihyperglycemic mechanism carried out by the behavior of γ‐conglutin in the face of insulin receptor and insulin, suggesting that there are two action levels, one mediated by lectin character as activity is needed for binding to membrane receptors and the other by the primary structure of γ‐conglutin, as native structure is not mandatory to insulin binding. Physiologically, that can be extremely relevant since, after digestion, if γ‐conglutin is degraded, its activity could be maintained.

In light of these results, different interpretations can be suggested to explain the antihyperglycemic effect obtained for purified γ‐conglutin. One of them could be the improvement of receptor sensitivity to insulin or to the complex insulin‐γ‐conglutin, extending, for example, the contact time with the receptor, thus promoting an enhanced glucose reuptake. Another possible interpretation relates to the possibility of γ‐conglutin connecting to the glycan moiety of insulin receptor with a higher affinity than that of human insulin. Pancreatic biomarker evaluation added new information, revealing that both native and denatured γ‐conglutin (lectin activity) enhanced insulin levels, matching the values obtained for metformin (Figure 7a). Although the present data do not allow for a full enlightenment of this effect, it suggests that this activity is one of the multiple mechanisms explaining the results obtained.

Lectins exhibit two types of mechanisms for binding glycosylated receptors, an extrinsic binding that can be identified by new polypeptide bands (1D) or spots(2D) in the original profile of HepG2 cell membranes, or/and by an intrinsic binding mechanism with internalization (Ribeiro et al., 2018; Fu et al., 2011) via mitochondria. In this study, only the extrinsic mechanism is evidenced, although the intrinsic mechanism can also occur. The internalization of γ‐conglutin has already been described as the multiple phosphorylation suffered by this molecule (Capraro et al., 2013), suggesting that both mechanisms participate in hypoglycemic activity.

From the point of view of glucose transporters, insulin binding to cell membrane receptor initiates a series of cascading phosphorylation/dephosphorization reactions, which results in cellular metabolic changes, necessary after binding of this hormone to cells to increase glucose absorption by, as well as expression, recruitment and translocation of glucose transporters such as GLUT2 (Ohtsubo et al., 2013) and GLUT4 (Saltiel & Kahn, 2001). It has been reported that cell stimulation (rat myoblasts) with γ‐conglutin led to decreased blood glucose and to the recruitment and translocation of GLUT4 (the primary effects of binding insulin to its membrane receptor) (Terruzzi et al., 2011). The glucose transporters are also glycosylated molecules with a single N‐linked oligosaccharide (Mueckler & Thorens, 2013). Concerning GLUT2 in hepatic tissue, it is the most expressed in beta cells, basolateral surface of kidney, hepatocytes, and small intestine epithelia (Orci et al., 1990; Thorens et al., 1990), with expression being regulated by sugars and hormones (Leturque et al., 2009). Patients with diabetes mellitus and with prolonged hepatitis C virus (HCV) infection have been related to viral‐induced reduction in hepatocyte expression of GLUT2 (Kasai et al., 2009), which exhibit an N‐glycan moiety for which GnT‐IV, a glycosyltransferase is required. GLUT2‐mediated glucose sensing is essential for maintaining normal glucose‐stimulated insulin secretion in pancreatic beta cells. The N‐glycan structure acts as a ligand for galectins to form the glycan–galectin lattice that maintains the stable cell surface expression of GLUT2, and cellular glucose transport activity (Ohtsubo et al., 2013). Knowing that galectins are lectins and are β‐galactoside‐binding proteins sharing homology in the amino acid residue sequence of their carbohydrate‐recognition domain, and that several glycan ligand recognition in GLUT exists for galectins, as well as disaccharide Galβ1‐4GlcNAc and other substrates with substitution at O‐2 and O‐3 of galactose residue, as well as core fragments ("right" from GlcNAc), that provides significant increase in affinity (Rapoport & Bovin, 2015), we could hypothesize that γ‐conglutin may establish a competition binding mechanism with galectins in order to bind to N‐glycan structure of glucose transporters that exhibit galactose domains, resulting in an antihyperglycemic effect.

The in vivo assays provided plausible information by dose‐response about different molecular targets involved in the hypoglycemic effect observed in animal models, after γ‐conglutin exposure. Previous works in hyperglycemic animal models reported that γ‐conglutin decreased plasma and serum glucose levels (González‐Santiago et al., 2017; Lovati et al., 2011; Magni et al., 2004; Sandoval‐Muñíz et al., 2018; Terruzzi et al., 2011), with exhibition of some molecular targets as it was evidenced by Vargas‐Guerrero et al. (2014), when STZ animal models exposed with γ‐conglutin showed benefic effects due to reductions in glucose, increments in serum insulin, and increases in Ins‐1 gene expression and beta cell insulin content compared with the STZ control group. A recent study (Sandoval‐Muñíz et al., 2018) described that after γ‐conglutin administration to streptozotocin‐induced rats, a slight increase in Slc2a2 and Pdx‐1 mRNA levels in pancreas and up‐regulated Slc2a2 expression in the liver were observed, but with no effect on hepatic glucokinase (GCK) expression, and with normalized GLUT2 protein content in pancreas of the of streptozotocin‐induced rats (Sandoval‐Muñíz et al., 2018). Also, modification of the gene expressions of enzymes G6pc and Fbp1 (fructose‐bisphosphatase 1 gene), involved in glucose hepatic production in vivo, and also Pck1 (phosphoenolpyruvate carboxykinase 1 gene) was studied in two experimental animal models of impaired glucose metabolism (insulin resistance IR and STZ models), where, after γ‐conglutin treatment, G6pc (glucose‐6‐phosphatase gene) expression was decreased in the IR‐γ‐conglutin and STZCγ groups. Post‐treatment, Fbp1 and Pck1 expressions were reduced in the IR‐γ‐conglutin group but increased in STZ‐γ‐conglutin animals. The authors suggested that γ‐conglutin is involved in reducing hepatic glucose production, mainly through G6pc inhibition in impaired glucose metabolism disorders (González‐Santiago et al., 2017).

In this work, the animal groups were established to test the antihyperglycemic activity of different structural forms of γ‐conglutin, in order to produce reliable and robust results, in agreement with the 3Rs policy. The results obtained in this hyperglycemic male mouse model aimed to evaluate serum glucose levels by the use of glucose oral tolerance test (GOTT) and the measurement of organ injury/functional biomarkers.

Mice were administrated by oral gavage with a dose of 60 mg/kg BW, selected according to previous reports (Magni et al., 2004), of both native γ‐conglutin with lectin activity and denatured γ‐conglutin. GOTT results showed that both conglutin forms exhibited antihyperglycemic effect although the denatured form originated better results than the native γ‐conglutin (exhibiting lectin activity), evidenced 30 min after glucose administration, whereas glucose values after 120 min were the same for all hyperglycemic groups (untreated and treated). The antihyperglycemic effect of γ‐conglutin was already reported; however, it is the first time that the primary structure of γ‐conglutin, rather than its native form, is shown to be one key factor for its antihyperglycemic activity, leading to the interpretation that, after physiological digestion, its effectiveness remains. It is possible that both γ‐conglutin forms exhibit antihyperglycemic activities, which are established by different mechanisms. Native γ‐conglutin has lectin activity, and in this state, it could bind to cell membrane‐glycosylated receptors, producing a mimetic effect of insulin in glucose reuptake. Also, a mechanism similar to the galectins could be responsible for binding to glucose transporters stimulating insulin secretion in pancreatic beta cells. The denatured form of γ‐conglutin could exhibit an antihyperglycemic activity by a mechanism that could be explained by binding to insulin, although this result needs to be further studied.

Several serum biomarkers with importance in the screening of T2D were evaluated to understand the behavior of *Lupinus* conglutins in this etiology, ruling out possible safety issues. For the pancreatic function evaluation, the biomarkers studied were insulin, amylase, and lipase. For amylase and lipase (Figure 7a,b) all animal groups exhibited similar results to those obtained for the normoglycemic group, with the exception for insulin values (Figure 7c), where the metformin group and both conglutins showed higher values (around 8.5 mU/l) than the hyperglycemic group (7m U/l), with this group showing a great increment compared with the normoglycemic group (1.5 mU/l). These markers reflect the normal state of the pancreas, and these results indicate the lack of toxicity to the molecules used in the assay, highlighting the increase in insulin production at a glucose overload, comparable to the result obtained for metformin. Regarding biomarkers for hepatic function, the ALT and AST results are presented in Figure 8 (a,b). For both biomarkers, marginal changes, although significant, were observed in serum levels of the hyperglycemic group when compared to the normoglycemic group. In general, both conglutin forms were able to attenuate those changes, with results not statistically different from the normoglycemic group. The metformin group was the one that had higher variation of these enzymes when compared to the normoglycemic group, although the magnitude of the changes is small and does not necessarily indicate any toxic effect by rather a consequence of direct pharmacodynamics effects in the liver function.

The effect on renal function by measurement of creatinine and urea is presented in Figure 9a,b. Induction of hyperglycemia led to a reduction in creatinine levels, although given the magnitude of the effect, it can be assumed to be a transitory physiological compensation without specific relevance. Other than the Cγ‐Nat group (in which no significant change was observed compared with the normoglycemic group), all other groups exhibited the same pattern of reduction. For urea, all groups exhibited no significant differences, as all groups originated results comparable to the normoglycemic group (between 34–38 U/L).

For total cholesterol (TC), HDL, LDL, and triglycerides (TG), regarding lipid profile evaluation, denatured γ‐conglutin (Cγ‐DT) was the experimental group that, in general, was associated with less variability in lipid profile, when compared to the normoglycemic group. The result with the highest magnitude of change was associated with the increase in LDL‐cholesterol levels in the hyperglycemic group, and increase that was inhibited by all experimental groups. Although these markers were performed solely with the aim of clarifying preliminary effects in several physiological parameters, specific studies in hypercholesterolemic models might enlighten some of the results obtained and complement already existing literature regarding the effect of *Lupinus albus* in dyslipidemia (Sirtori et al., 2004).

## 5 CONCLUSIONS

This work reveal that in the modulation of diabetes exerted by γ‐conglutin, several mechanisms are involved in order to regulate blood glucose levels. The display of lectin activity, associated with the native structure, as well as the activity shown by the primary structure of γ‐conglutin, could be reflected in its antihyperglycemic activity. These capacities were involved in the binding of native **γ**‐conglutin to HepG2 cell glucose receptors, including insulin receptors, as well as their binding to human insulin, but also with the ability of denatured **γ**‐conglutin to bind to insulin.

The skills of **γ**‐conglutin give a versatility in its action mechanism. These skills were evidenced in in vivo experiments where the denatured form of **γ**‐conglutin had better results than native form, at OGTT (oral glucose tolerance test) and in pancreatic, hepatic, and dyslipidemic biomarkers. Also, the native **γ**‐conglutin reveals its antihyperglycemic effect, which seems to be necessary to exhibit its lectin activity effect.

In conclusion, native **γ**‐conglutin with lectin activity and denatured **γ**‐conglutin seem to have therapeutic potential, sometimes can rival with the institutional drug metformin.

## CONFLICT OF INTEREST

The authors declare no conflict of interest to publish the results.

## AUTHOR CONTRIBUTIONS

M.G. performed the vast majority of the experiments, helped by A.C.R.; J.R. realize the in vivo experiments helped by J.S. and R.P. realize the serological assays; R.B.F. and E.F. give the resources for execute the project; R.B.F., J.R. and B.S. commented on the manuscript and provided essential feedback; A.C.R. do the conceptualization of the project, supervised the project helped by R.B.F. and draft and prepare the final version of the manuscript (helped by M.G. and R.B.F.) and editing. Conceived and design the experiments: A.C.R. and J.R.
